# Correlation analysis between semen routine parameters and sperm DNA fragmentation index in patients with semen non-liquefaction: A retrospective study

**DOI:** 10.1515/biol-2022-1020

**Published:** 2025-05-20

**Authors:** Fengqing Ji, Junying Chen, Liyun Lin

**Affiliations:** Department of Clinical Laboratory, Xiamen Hospital of Traditional Chinese Medicine, 1739 Xianyue Road, Huli District, Xiamen, Fujian 361009, China; Department of Clinical Laboratory, Xiamen Hospital of Traditional Chinese Medicine, Xiamen, Fujian 361009, China

**Keywords:** infertility, semen non-liquefaction, sperm DNA fragmentation, routine semen parameters

## Abstract

This research investigated the correlation between routine semen parameters and the sperm DNA fragmentation index (DFI) in infertile males with non-liquefying semen, aiming to provide insights for infertility management. We conducted a retrospective analysis of 152 infertile males at our Andrology Department from March 2023 to March 2024, assessing differences in semen parameters and DFI based on liquefaction times and identifying influential factors on DFI. Participants were divided into two groups based on their semen liquefaction times: less than 60 min (111 patients) and 60 min or more (41 patients). The group with normal liquefaction times demonstrated significantly better forward and total sperm motility, and lower sperm morphology indices and DFI values. A positive correlation was found between DFI and liquefaction time, while a negative correlation was observed with motility and normal sperm ratio. Specifically, each additional minute of liquefaction time increased DFI by 0.13, and a 1% decrease in normal sperm ratio increased DFI by 0.73. The findings indicate that delayed semen liquefaction correlates with poorer sperm motility and morphology and higher DFI, underscoring the importance of comprehensive semen assessment in evaluating male fertility.

## 1 Introduction

Approximately 15% of couples in their reproductive age experience infertility, defined as the inability to conceive after 1 year of unprotected intercourse. Male factors contribute to about half of these case, with semen quality being a critical determinant of male fertility, affected by various environmental, lifestyle, and age-related factors [1]. These influences can lead to abnormal sperm morphology, reduced semen volume, and decreased sperm motility. Notably, recent studies have documented a decline in semen quality among Chinese men [2].

Globally, conventional semen analysis is considered the gold standard for diagnosing male infertility. The diagnostic method assesses various parameters, including semen appearance, volume, pH, liquefaction time, sperm concentration, and total sperm count [3]. However, it has limitations in accurately evaluating sperm functionality. Notably, studies have indicated that approximately 15% of infertile men exhibit semen parameters that fall within the normal ranges established by the World Health Organization [4], suggesting defects of the current diagnosis.

The sperm DNA fragmentation index (DFI) has emerged as a novel marker for assessing sperm function, indicating the integrity of sperm DNA and the extent of DNA damage during spermatogenesis and sperm maturation. DFI is crucial for evaluating male fertility potential and is strongly associated with adverse pregnancy outcomes [5]. Although there are ongoing debates about its routine use, the diagnostic value of DFI is recognized by both the American Urological Association and the European Association of Urology [5]. A comprehensive assessment that incorporates both conventional semen parameters and DFI provides a more complete evaluation of a patient’s semen status, including sperm vitality, morphology, genetic integrity, and accessory gland function, which are essential for accurately diagnosing and managing infertility [6,7].

Semen liquefaction time – the period needed for semen to transition from a gelatinous to a liquid state – typically ranges from 20–30 min. Semen that does not liquefy within 60 min is considered to exhibit non-liquefaction, which may indicate issues with sperm quality or function [8]. In such instances, sperm can become trapped in the coagulum, impeding motility and passage through the cervix, and increasing the likelihood of sperm death due to prolonged exposure in the reproductive tract [9].

We hypothesize that semen non-liquefaction is associated with increased sperm DNA fragmentation, contributing to reduced male fertility. This study aims to evaluate the correlation between routine semen parameters and the sperm DFI in infertile males with non-liquefying semen, to provide insights into the implications of non-liquefaction on male fertility and to develop more nuanced diagnostic approaches.

## 2 Materials and methods

### 2.1 Study design

A retrospective analysis was conducted on a cohort of infertile males admitted to our Andrology Department between March 2023 and March 2024, focusing on their clinical data.

### 2.2 Inclusion and exclusion criteria

#### 2.2.1 Inclusion criteria

1. Married males aged 20–45 years.
2. Diagnosed with infertility, defined as the inability to conceive after 1 year of unprotected intercourse with no female factor infertility identified.
3. Negative for *Mycoplasma*, *Chlamydia*, and *Neisseria gonorrhoeae*.
4. Semen liquefaction time exceeding 60 min.

#### 2.2.2 Exclusion criteria

1. Presence of urological disorders such as varicocele, cryptorchidism, prostatitis, epididymitis, hematospermia, or azoospermia.
2. History of reproductive tract injury.
3. Recent use of medications known to affect semen quality (within the last month).
4. Presence of concurrent malignancy or hematological disorders.

**Informed consent:** Informed consent has been obtained from all individuals included in this study.
**Ethical approval:** The research related to human use has been complied with all the relevant national regulations, institutional policies and in accordance with the tenets of the Helsinki Declaration, and has been approved by the authors’ institutional review board or equivalent committee.

### 2.3 Semen analysis

Semen samples were collected via masturbation after 2–7 days of sexual abstinence, as per WHO guidelines. The samples were then liquefied at 37°C, and a 5 μL aliquot was used for microscopic enumeration on a disposable hemocytometer slide, assessing parameters including semen volume, total sperm count, sperm concentration, sperm motility (progressive, non-progressive, immotile), liquefaction time, and pH. These parameters were quantitatively assessed using an SCA Sperm Analyzer (Spain), with established normal reference ranges: semen volume ≥1.5 mL, sperm concentration ≥15 × 10^6^/mL, progressive motile sperm (PR) ≥32%, and total motility ≥40%.

### 2.4 Sperm morphology assessment

Sperm morphology was evaluated using the Papanicolaou staining method, where a technician examined at least 200 sperm per slide under a 100× oil immersion lens and a 10× ocular lens, identifying defects in the head, midpiece, and tail. The sperm deformity index (SDI) was calculated according to the WHO 5th edition guidelines.

### 2.5 Sperm DFI detection and grouping

Semen samples were sent to a third-party laboratory for sperm DFI testing using a Sparrow flow cytometer and Selena reagent kit. The analysis adjusted sperm concentration to 1 × 10^6^–2 × 10^6^ cells/mL. A 100 μL aliquot was analyzed for at least 5,000 sperm using flow cytometry and the sperm chromatin structure assay, exploiting acridine orange’s metachromatic properties to differentiate intact double-stranded DNA (green fluorescence) from fragmented single-stranded DNA (red fluorescence). Patients were categorized into three groups based on DFI: ≤15.00% (good sperm DNA integrity), 15.00 to <30.00% (moderate sperm DNA integrity), and ≥30.00% (poor sperm DNA integrity).

### 2.6 Sample size justification

The target sample size was calculated to detect a medium effect size (Cohen’s *d* = 0.5) with a power of 80% and a significance level of 0.05, which initially suggested a minimum of 64 participants per group. However, due to the lower than expected prevalence of non-liquefaction among our patient population during the study period, only 41 cases were included in the Delayed Liquefaction group. To mitigate this limitation, we increased the overall sample size to 152 participants to enhance the study’s statistical power. Additionally, we employed robust statistical techniques to maximize the use of the available data and ensure that our findings remained statistically significant despite the smaller subgroup size. The difference in group sizes was further accounted for in our statistical analysis, adjusting for potential biases that could arise from the uneven distribution.

### 2.7 Statistical analysis

Statistical analyses were conducted using SPSS 25.0 and the R programming language. Continuous data were expressed as mean ± standard deviation and compared using *t*-tests, while categorical data were reported as percentages and analyzed using chi-square tests. The relationships between DFI and routine semen parameters were assessed via Pearson correlation analysis, defining the strength of correlation as follows: |*r*| > 0.9 (very strong), 0.7 < |*r*| < 0.9 (strong), 0.4 < |*r*| < 0.7 (moderate), 0.2 < |*r*| < 0.4 (weak), and 0 < |*r*| < 0.2 (very weak or none). Statistical significance was established at *P* < 0.05, using a two-tailed approach.

## 3 Results

### 3.1 Semen parameters and DFI based on different semen liquefaction times

In this study, we examined 152 infertile males who met the inclusion criteria. These participants were divided into two groups according to their semen liquefaction times: a normal liquefaction group (liquefaction time <60 min, 111 cases) and a delayed liquefaction group (liquefaction time ≥60 min, 41 cases). Table 1 displays a comparative analysis of routine semen parameters and the sperm DFI between these groups.

**Table 1:** Semen parameters and DFI based on different semen liquefaction times

	Normal liquefaction group (*n* = 111)	Delayed liquefaction group (*n* = 41)	*t*/*χ* ^2^	*P*
Age (years)	34.26 ± 6.33	36.21 ± 7.15	1.669	0.097
Semen volume (mL)	3.57 ± 1.43	3.36 ± 1.14	0.846	0.399
Sperm concentration (×10^6^/mL)	57.36 ± 20.96	52.39 ± 17.36	1.355	0.177
PR (%)	41.36 ± 11.75	29.17 ± 5.33	6.394	<0.001
Sperm vitality (A + B) (%)	61.36 ± 19.36	40.11 ± 11.93	6.574	<0.001
Teratozoospermia index	1.42 ± 0.07	1.97 ± 0.12	34.91	<0.001
Normal morphology (%)	4.37 ± 1.74	3.93 ± 1.61	1.411	0.160
DFI	21.16 ± 5.36	32.31 ± 8.31	9.710	<0.001

The age distribution was similar between both groups, indicating that age was not a confounding factor in the analysis. Notably, the normal liquefaction group exhibited significantly higher levels of progressive motility and overall sperm motility compared to the delayed liquefaction group. Furthermore, this group also showed a lower SDI and decreased levels of DFI, with these differences being statistically significant (*P* < 0.05).

### 3.2 Comparison of abnormal semen parameters rates at different DFI levels

Within this study, semen parameters were categorized as normal if they met the following criteria: semen volume ≥1.5 mL, sperm concentration ≥15 × 10^6^/mL, progressive motility (PR) ≥32%, and total sperm motility ≥40%. The 152 infertile patients were further stratified based on their sperm DFI levels into three groups: DFI ≤ 15%, 15% < DFI < 30%, and DFI ≥ 30%. The prevalence of abnormal semen parameters among these groups is detailed in Table 2.

**Table 2:** Comparison of abnormal semen parameters rates at different DFI levels

	Total	Normal semen parameters	Abnormal semen parameters
Total	152	121	31
DFI ≤ 15%	59	17	42
15% < DFI < 30%	52	12	40
DFI ≥ 30%	41	2	39

Out of the total participants, 31 exhibited normal semen parameters. Distribution within the DFI categories was as follows: 17 patients in the DFI ≤ 15% group, 12 in the 15% < DFI < 30% group, and only 2 in the DFI ≥ 30% group. Significant differences were observed in the rates of abnormal semen parameters across these DFI levels. The groups with DFI ≤ 15% and 15% < DFI < 30% had considerably lower rates of abnormal parameters compared to the DFI ≥ 30% group. However, no significant difference was noted between the DFI ≤ 15% and 15% < DFI < 30% groups.

### 3.3 Correlation analysis between DFI and semen parameters

**Figure 1:**
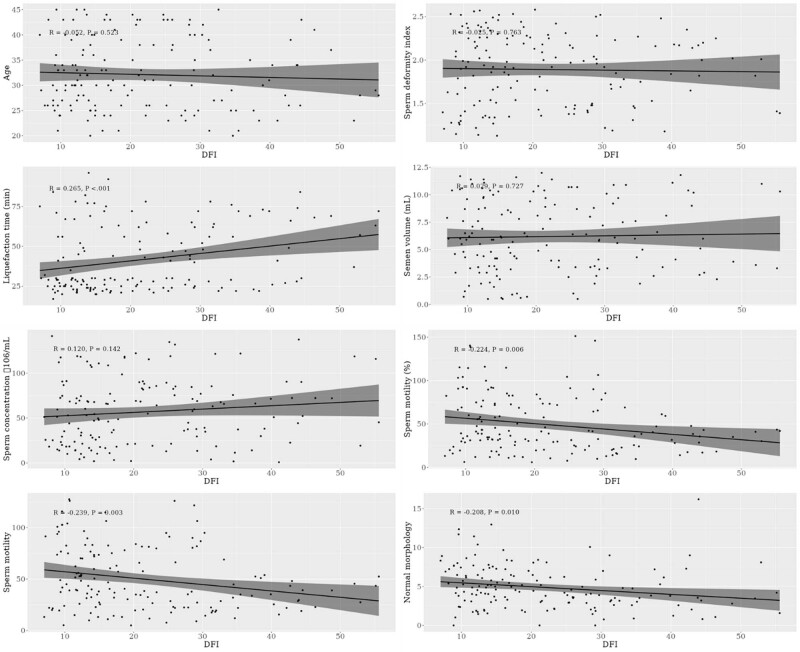
Correlation analysis between DFI and semen parameters.

To further elucidate the relationship between sperm DFI and key semen characteristics, correlation analyses were conducted. In this analysis, DFI was treated as the dependent variable, while the following semen parameters served as independent variables: semen liquefaction time, sperm volume, sperm concentration, progressive motility (PR), total sperm motility, sperm morphology index, and the ratio of normal sperm (illustrated in Figure 1).

The results indicated a positive correlation between DFI and semen liquefaction time, implying that longer liquefaction times are associated with increased DNA fragmentation in sperm. Conversely, negative correlations were observed between DFI and several other parameters: progressive motility (PR), overall sperm motility, and the ratio of morphologically normal sperm. These findings suggest that higher levels of DFI are linked to reduced sperm motility and a lower proportion of morphologically normal sperm, highlighting the detrimental impact of compromised DNA integrity on sperm function and quality.

### 3.4 Univariate linear regression analysis of DFI

To further explore the relationship between sperm DFI and semen parameters, both univariate and multivariate linear regression analyses were performed. In these analyses, DFI was established as the dependent variable, with predictors including age and various semen parameters.

The univariate linear regression analysis revealed significant linear correlations between DFI and key semen parameters such as semen liquefaction time (*p* < 0.001), progressive motility (PR) (*p* = 0.02), total sperm motility, (*p* = 0.06) and the ratio of normal sperm (*p* = 0.010) (results detailed in Table 3). These findings indicate that certain semen parameters can serve as predictors of the extent of DNA fragmentation in sperm. This analysis provides valuable insights into the potential mechanisms influencing sperm quality and fertility outcomes, suggesting that alterations in these parameters could be indicative of underlying reproductive health issues.

**Table 3:** Univariate linear regression analysis for DFI

Variables	*b*	S.E	*t*	*P*	*β* (95% CI)
Age	−0.09	0.14	−0.64	0.523	−0.09 (−0.35 to 0.18)
Liquefaction time (min)	0.15	0.04	3.37	**<0.001**	0.15 (0.06 to 0.24)
Semen volume (mL)	0.10	0.29	0.35	0.727	0.10 (−0.47 to 0.68)
Sperm concentration (×10^6^/mL)	0.04	0.03	1.47	0.142	0.04 (−0.01 to 0.09)
PR (%)	−0.13	0.04	−3.19	**0.002**	−0.13 (−0.21 to −0.05)
Sperm motility (%)	−0.08	0.03	−2.81	**0.006**	−0.08 (−0.14 to −0.02)
SDI	−0.70	2.33	−0.30	0.763	−0.70 (−5.27 to 3.86)
Normal morphology	−0.87	0.34	−2.60	**0.010**	−0.87 (−1.53 to −0.21)

CI: confidence interval.

### 3.5 Multivariate linear regression analysis

A multivariate linear regression analysis was employed to develop a predictive model for the sperm DFI, with DFI considered as the dependent variable (*Y*). The predictors included in the model were semen liquefaction time (*X*1), progressive motility (PR) (*X*2), sperm motility (*X*3), and the ratio of normal sperm (*X*4). The resulting model equation is as follows:

DFI (*Y*) = 0.13(*X*1) − 0.73(*X*4) + 23.85.

This model elucidates the relationships between DFI and the chosen predictors. Specifically, the analysis reveals that each 1 min increase in semen liquefaction time is associated with a 0.13 unit increase in DFI (*p* = 0.004). Additionally, a 1% decrease in the normal sperm ratio results in a 0.73 unit increase in DFI (*p* = 0.025). These findings, summarized in Table 4, underscore the significant influence of semen liquefaction time and the quality of sperm on the level of DNA fragmentation. This information provides critical insights for clinical evaluations and developing treatment strategies for male infertility.

**Table 4:** Multivariate linear regression analysis for DFI

Variables	*b*	SE	*t*	*P*	*β* (95% CI)
Intercept	23.85	3.12	7.65	**<0.001**	23.85 (17.74 to 29.97)
Liquefaction time (min)	0.13	0.04	2.90	**0.004**	0.13 (0.04 to 0.22)
PR (%)	−0.08	0.10	−0.81	0.422	−0.08 (−0.29 to 0.12)
Sperm motility (%)	−0.01	0.07	−0.21	0.834	−0.01 (−0.15 to 0.12)
Normal morphology	−0.73	0.32	−2.26	**0.025**	−0.73 (−1.37 to −0.10)

CI: confidence interval.

## 4 Discussion

This study highlights a significant association between delayed semen liquefaction and impaired sperm quality in infertile men, with delayed liquefaction observed in approximately 12% of infertility cases [9]. Semen liquefaction, facilitated by SEMG1 and SEMG2 proteins from the seminal vesicles, is crucial for sperm release and fertilization. These proteins form a gel-like barrier that encapsulates sperm post-ejaculation, which is then broken down by prostate-specific antigen or KLK3, enhancing sperm motility for successful fertilization [10–12]. Our findings suggest that optimizing liquefaction times could markedly improve fertility outcomes, underscoring the importance of further research into the mechanisms and treatment of delayed liquefaction to boost reproductive success in affected individuals.

Genetic variations, biochemical disruptions, and pathological conditions affecting the male accessory organs are significant contributors to defects in semen liquefaction processes [13]. Under microscopic examination, non-liquefied semen exhibits a fibrous net structure that impedes sperm movement, thus reducing motility and increasing reproductive challenges [8].

Our findings reveal clear disparities in semen parameter abnormalities across different DFI levels. Groups with DFI ≤ 15% and 15% < DFI < 30% demonstrated significantly lower rates of abnormal parameters compared to the DFI ≥ 30% group. Correlation analyses showed a positive association between DFI and semen liquefaction time, and negative correlations with progressive motility (PR), total sperm motility, and the ratio of normal sperm. Our multivariate regression model further highlighted these relationships, indicating that an increase in DFI is associated with prolonged liquefaction time and a reduced ratio of normal sperm. Such sperm damage is often due to factors like improper chromatin packing, abnormal sperm apoptosis, and oxidative stress, which also influence seminal vesicle secretions – such as coagulation factors and fibronectin – that affect liquefaction time [14,15].

Our findings are consistent with previous research. For example, L-carnitine treatments have been shown to reduce DFI and improve progressive motility (PR) [16]. Similarly, prior studies confirmed negative correlations between DFI and critical semen parameters such as sperm concentration and motility [17,18]. Additionally, the presence of leukocytes in semen, which release reactive oxygen species during phagocytosis, contributes to DNA damage and is associated with increased DFI, illustrating the role of oxidative stress in affecting semen quality [19]. Moreover, viscous semen, which induces oxidative stress, correlates with poor sperm quality and increased DFI, leading to semen non-liquefaction [20].

The study’s limitations include its small sample size and cross-sectional design, which may limit the generalizability of the findings and prevent the establishment of causality. Future research would benefit from a larger and more diverse sample to improve representativeness and from using longitudinal designs to better explore temporal relationships and causality. This study focused primarily on the relationship between delayed liquefaction and basic semen parameters; however, further research should also consider other influencing factors such as genetic variations, biochemical disruptions, and pathological conditions of male accessory organs. Moreover, controlling for confounding variables like age, lifestyle, comorbidities, and medication use will be crucial for enhancing the accuracy and reliability of the results. Expanding these research areas will provide deeper insights into the complex dynamics influencing male fertility and semen quality.

Our findings highlight a significant correlation between prolonged semen liquefaction times and increased sperm DFI, suggesting that DFI should be considered in routine infertility assessments. This could lead to more targeted and effective treatments for infertile males, particularly those with non-liquefying semen. Integrating DFI assessments into clinical practice could also aid clinicians in identifying cases where genetic integrity of sperm is compromised, potentially guiding more personalized fertility treatments. The variability in semen quality and DFI observed globally suggests that regional healthcare systems might need to adapt our findings differently. Developing countries, where access to specialized reproductive health services may be limited, could benefit from basic interventions aimed at improving general health and lifestyle factors that contribute to better semen quality. Meanwhile, in more developed regions, advanced diagnostic and treatment options could be integrated into existing healthcare frameworks to address infertility more effectively.

In conclusion, this study has established significant associations between delayed semen liquefaction in infertile males and several adverse semen quality parameters, including decreased progressive motility (PR), reduced total sperm motility, increased abnormal sperm morphology, and elevated DFI. These findings underline the importance of a comprehensive assessment of male fertility potential, integrating both physical semen characteristics and molecular markers of sperm integrity. Understanding these interrelationships is crucial for the accurate diagnosis and effective treatment of male infertility, highlighting the need for multifaceted approaches in clinical settings.
